# A universal assay for detection of oncogenic fusion transcripts by oligo microarray analysis

**DOI:** 10.1186/1476-4598-8-5

**Published:** 2009-01-19

**Authors:** Rolf I Skotheim, Gard OS Thomassen, Marthe Eken, Guro E Lind, Francesca Micci, Franclim R Ribeiro, Nuno Cerveira, Manuel R Teixeira, Sverre Heim, Torbjørn Rognes, Ragnhild A Lothe

**Affiliations:** 1 Department of Cancer Prevention, Institute for Cancer Research, Norwegian Radium Hospital, Rikshospitalet University Hospital, Oslo, Norway; 2 Centre for Cancer Biomedicine, University of Oslo, Oslo, Norway; 3 Centre for Molecular Biology and Neuroscience, Institute of Medical Microbiology, Rikshospitalet University Hospital, Oslo, Norway; 4 Department of Molecular Biosciences, University of Oslo, Oslo, Norway; 5 Department of Cancer Genetics, Norwegian Radium Hospital, Rikshospitalet University Hospital, Oslo, Norway; 6 Department of Genetics, Portuguese Oncology Institute, Porto, Portugal; 7 Medical Faculty, University of Oslo, Oslo, Norway; 8 Department of Informatics, University of Oslo, Oslo, Norway

## Abstract

**Background:**

The ability to detect neoplasia-specific fusion genes is important not only in cancer research, but also increasingly in clinical settings to ensure that correct diagnosis is made and the optimal treatment is chosen. However, the available methodologies to detect such fusions all have their distinct short-comings.

**Results:**

We describe a novel oligonucleotide microarray strategy whereby one can screen for all known oncogenic fusion transcripts in a single experiment. To accomplish this, we combine measurements of chimeric transcript junctions with exon-wise measurements of individual fusion partners. To demonstrate the usefulness of the approach, we designed a DNA microarray containing 68,861 oligonucleotide probes that includes oligos covering all combinations of chimeric exon-exon junctions from 275 pairs of fusion genes, as well as sets of oligos internal to all the exons of the fusion partners. Using this array, proof of principle was demonstrated by detection of known fusion genes (such as *TCF3:PBX1*, *ETV6:RUNX1*, and *TMPRSS2:ERG*) from all six positive controls consisting of leukemia cell lines and prostate cancer biopsies.

**Conclusion:**

This new method bears promise of an important complement to currently used diagnostic and research tools for the detection of fusion genes in neoplastic diseases.

## Background

Fusion genes created by structural chromosomal rearrangements such as translocations, deletions, and inversions are often the pathogenetically essential feature of cancer genomes. They seem to be particularly characteristic of hematological malignancies and sarcomas, where their identification may be crucial for differential diagnosis and therapeutic decision-making. Fusion genes have so far been found less frequently in the common solid cancers, but recent reports on prostate and lung carcinomas show that fusion transcripts may contribute significantly also to the development of these malignancies [refs. [1-3]; reviewed in [4,5]].

The detection of fusion genes in cancer is laborious and time-consuming and usually includes chromosome banding analysis (karyotyping) followed by fluorescence *in situ *hybridization (FISH) studies and molecular analyses based on the reverse transcriptase polymerase chain reaction (RT-PCR). Karyotyping requires the availability of fresh, vital cells for short-term culturing to obtain metaphase chromosomes, and the success rate of this approach may be particularly low for solid tumors. In addition to taking a lot of time, the method also requires highly trained and experienced personnel to interpret the karyotypes correctly and identify whatever rearrangements may exist. The main advantage of the approach is that it is global in nature; it screens without prejudice for all rearrangements at the chromosomal resolution level. FISH with locus-specific probes and RT-PCR, on the other hand, are precise and highly specific methods used for the analysis of one or a few candidate fusion genes at predefined breakpoints; the approach is therefore dependent on prior knowledge of the suspected diagnosis. The specificity of these methods at the same time highlights their main limitation; they have no screening ability.

Recent developments of high-throughput sequencing technologies enable genome-wide identification of novel fusion transcripts at an unprecedented level of resolution [6-9], but these technologies are as yet limited by the number of samples that can be analyzed within a reasonable time-frame and at an acceptable cost. A few studies have utilized oligo microarrays targeting junction sequences to detect fusion transcripts [10-13]. They have then relied on preceding amplification of a small selection of fusion transcripts by RT-PCR, thus limiting the coverage offered by these approaches to a predefined set of fusion junction sequences.

In this report, we present a new oligo microarray-based approach for simultaneous analysis of all known or predicted fusion gene variants, with all possible chimeric exon-exon junction combinations. The analysis can be performed in a single experiment and does not include prior sequence-specific amplification.

## Methods

### Cell lines and biopsies

To test our novel method for fusion gene detection, we selected four prostate cancer samples (fresh frozen tissue obtained from prostatectomy specimens of four independent patients) and two leukemic cell lines, all known to harbor a specific fusion gene. The cell lines, RCH-ACV [14] and REH [15,16], are of human B-cell precursor leukemia origin and were provided by Dr. Edith Rian.

### Preparation of cDNA for microarray analysis and RT-PCR

Total RNA was isolated using the Trizol reagent (Life Technologies, Rockville, MD, USA), and the RNA quality was evaluated by use of the Agilent 2100 Bioanalyzer (Agilent Technologies, Palo Alto, CA, USA). To enrich for messenger RNA, we used the RiboMinus kit (Invitrogen, Carlsbad, CA, USA) which subtracts ribosomal RNA from total RNA. To ensure detection of fusion junctions far away from the poly-A tail, the first strand cDNA was prepared by random priming to avoid the 3' end bias introduced by oligo-dT labeling. Double stranded cDNA was labeled and hybridized onto the oligo microarrays.

### Microarray design

We set up a database with a broad coverage of the reported fusion genes in cancer (351 to date), including information on which of the fusion partners are up- and downstream in the majority of the resulting fusion transcripts. See Additional file 1 for the identities and orientation of the 275 fusion genes included in the pilot microarray design. We used public genome sequence information from Biomart to extract the exon sequences of all listed transcript variants [17].

A script was written in the programming language Python for design of the oligos. For genes that constitute the 5' portion of fusion genes, we used the 3' end-sequences of the exons when constructing chimeric fusion junction oligos. For genes that are the 3' portion of fusion genes, we used the 5' start-sequences of the exons. Thus, for each fusion gene, we joined and listed all combinations of end-sequences and start-sequences. These chimeric sequences served as input for the design of chimeric fusion junction oligos, enabling detection of any breakpoint combination in the fusion genes. Chimeric oligos were constructed targeting all possible combinations of chimeric exon junctions between the up- and downstream partners of 275 known fusion genes. For a set of fusion genes, including the ones known to be present in the control samples, we extended the design to include four replicates of each of the exon-exon junctions, as well as altogether four extra control oligos for each exon-exon junction (oligos up- and down-shifted by two nucleotides as compared to the standard ones). Furthermore, a series of intragenic oligos were designed for measurements of longitudinal profiles of each of the fusion gene partners of altogether 115 genes, including all the positive control fusion genes. These were oligos targeting the start, mid, and end part of all exons and all introns, as well as oligos targeting the exon-exon, exon-intron, and intron-exon junctions. The exon-intron junctions and intron-exon junctions are also included among the single-gene oligos, as the pre-mRNA processing machinery may alter the splicing pattern following removal or introduction of cis-acting splicing regulatory sequences.

The constructed microarray included a design with 68,861 oligos, including 59,381 chimeric oligos (of which 55,482 were unique), which were synthesized onto custom-produced NimbleGen microarray slides (Roche NimbleGen, Inc., Madison, WI, USA). The chimeric oligos were designed to optimize for similar melting temperatures on each side of the junctions, thus reducing half-binder effects.

Two versions of the microarray were designed, differing as to the probe lengths. The set of shorter oligos, with lengths ranging from 34 to 40-mers, had a Tm optimum of 72°C. The set of longer oligos, with lengths ranging from 44 to 50-mers, had a Tm optimum of 75°C. All samples, except the REH cell line, were hybridized onto the short-oligo microarray, whereas the RCH-ACV and REH cell lines were hybridized onto the long-oligo microarray. The cell line RCH-ACV was analyzed by both microarray designs, and data from its positive control gene, *TCF3:PBX1*, demonstrated best performance of the short oligos due to substantial half-binder signals with the longer oligos (data not shown).

Because of the relatively short length of the sequences on each side of the junction, the binding may be sensitive to single nucleotide polymorphisms (SNPs). Thus, at known SNP positions, we created extra sets of probes, accounting for each of the SNP variants.

### Data preprocessing and annotation

Data preprocessed by NimbleGen were further normalized by dividing all individual probe intensity values for each of the samples by the median of the three leukemia cell lines. We normalized based on these three samples (instead of all samples) because when the majority of the samples contain the same fusion gene and breakpoint (*TMPRSS2:ERG, e1:e4*), normalizing on all samples would level out the appearance of this fusion event in the dataset.

All oligonucleotide probes were mapped to their one or two respective genomic loci. For each locus, the Ensembl identifiers for exon (ENSE), transcript (ENST), and gene (ENSG) identities were used.

Raw and processed data were deposited to the Gene Expression Omnibus public repository for microarray data [accession number GSE14435] according to the MIAME, minimum information about a microarray experiment, recommendations for recording and reporting microarray-based gene expression data [18].

### Automated scoring algorithm

Downstream fusion partners will generally have higher expression values for exons downstream of the fusion breakpoint. For each exon-exon junction of downstream fusion partner genes, two probabilities were calculated. One probability was based on a t-test for whether values from all upstream and all downstream exons are likely to belong to different populations. A second probability was based on a t-test for whether the values from the immediate up- and downstream exons are likely to belong to different populations.

A fusion score was calculated as the product of the normalized expression value for the chimeric oligo and the probabilities of the exon-exon junction of the corresponding position in the downstream fusion partner being a breakpoint in the longitudinal profile [Fusion score = Chimeric junction score * P(B-gene transcript) * P(B-gene exon)].

To keep the values within scale, the following thresholds were applied: when the normalized values for chimeric oligos were larger than 5, they were set to 5 (approximately 5 per 10,000 values). Similarly, when probabilities for a breakpoint in the longitudinal profiles were < 0.10, they were set to 0.10. When the values from the downstream exons were lower than the values from the upstream exons, the probability was set to 0.10 as well.

### Experimental validation of fusion transcript breakpoints

We used RT-PCR followed by DNA sequencing to validate the actual fusion junctions in the positive control fusion genes. The following primers were applied: *TCF3:PBX1*: *TCF3*, exon 15, forward, 5'-CACCCTCCCTGACCTGTCT-3', and *PBX1*, exon 3, reverse, 5'-TGCTCCACTGAGTTGTCTGAA-3'; yielding a chimeric fusion product of 218 basepairs. *ETV6:RUNX1*: *ETV6*, exon 5, forward, 5'-CACTCCGTGGATTTCAAACA-3', and *RUNX1*, exon 2, reverse, 5'-CGTGGACGTCTCTAGAAGGA-3'; yielding a chimeric fusion product of 204 basepairs. *TMPRSS2:ERG *[as published in ref. [19]]: *TMPRSS2*, exon 1, forward, 5'-TAGGCGCGAGCTAAGCAGGAG-3', and *ERG*, exon 6, reverse, 5'-CTGCCGCACATGGTCTGTAC-3'; yielding a chimeric fusion product of 597 basepairs. The PCR products were separated by gel electrophoresis in a 2% agarose gel. For all fusion genes, DNA was isolated from the appropriate PCR bands (MiniElute Gel Extraction kit, Qiagen Co., Valencia, CA, USA) and sequenced in both directions using the same primers as for the RT-PCR (ABI Prism 3730; Applied Biosystems, Foster City, CA, USA).

### Cytogenetics

Cell cultures from the leukemia cell lines were harvested for chromosome banding analysis. Chromosome preparations were made and G-banded using trypsin (DIFCO Laboratories, Detroit, MI, USA) and Leishman staining (BDH, Poole, England). For metaphase FISH, commercially available probes for the *TCF3:PBX1 *(*TCF3 *FISH DNA probe, split signal, DAKO Denmark A/S, Glostrup, Denmark) and *ETV6:RUNX1 *(dual color, Dual Fusion Translocation Probe Set; Vysis, Abbott Laboratories, Abbott Park, IL, USA) fusion genes were used. The denaturation and hybridization conditions as well as the subsequent detection procedures were in accordance with the manufacturers' protocols. Two hundred successive, whole, and single nuclei were examined through a Zeiss fluorescence microscope (Zeiss Axioplan, Oberkochen, Germany) for each FISH experiment.

## Results

**Figure 1:**
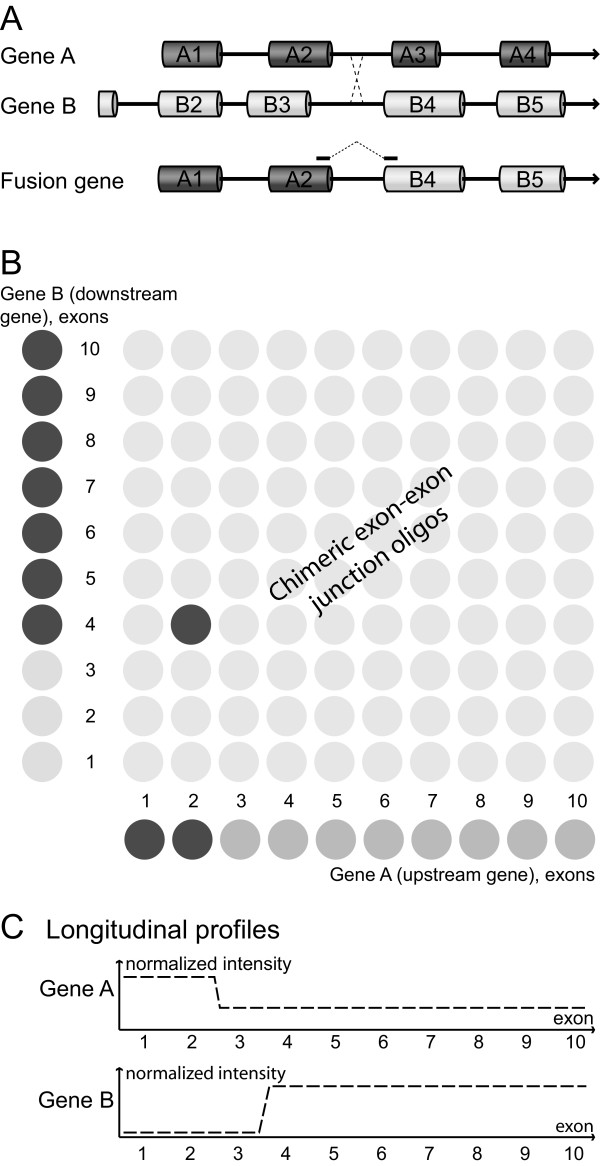
**Microarray data for a positive fusion gene hit**. (A) This theoretical example of a fusion gene has a crossing-over event between sequences in intron 2 of gene A and intron 3 of gene B. (B) If the genes A and B both have 10 exons, the microarray will contain 10 × 10 = 100 oligos to cover all chimeric exon-to-exon junction combinations for this particular fusion gene. The A2-B4 oligo detects the fusion transcript from part (A). (C) In true fusion events, the longitudinal profiles generated from intragenic oligos targeting each exon and exon-to-exon junction will provide independent confirmation.

We have developed a novel strategy for the detection of oncogenic fusion transcripts enabling simultaneous analysis of all known or predicted fusion gene variants, with all possible chimeric exon-exon junction combinations targeting each possible fusion gene junction on the processed mRNA level (Figure 1). We combine the use of chimeric oligos, spanning the two potential fusion gene partners, with the use of single-gene oligos that provide measurements along the length of each individual partner.

We analyzed cDNA from a set of six positive control samples with known presence of one fusion gene in each. This included two leukemia cell lines, RCH-ACV and REH, known to carry the *TCF3:PBX1 *and *ETV6:RUNX1 *fusion genes, respectively, and four prostate cancer samples positive for the *TMPRSS2:ERG *fusion gene.

**Figure 2:**
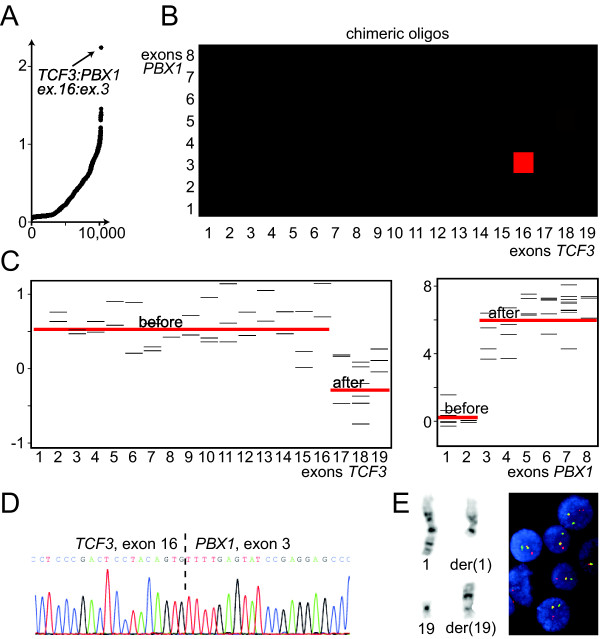
***TCF3:PBX1 *in a leukemia cell line**. (A) The highest ranking fusion score (y-axis) among 10,297 chimeric combinations (ranked along the x-axis) indicated a fusion event between exons 16 and 3 of *TCF3 *and *PBX1*, respectively. (B) Fusion map of each chimeric exon-exon junction of *TCF3 *and *PBX1*. Intensities of red indicate the relative values of the medians for the four replicate oligos for each chimeric exon-exon junction, and the square with strongest intensity indicates the correct fusion breakpoint. (C) Measurements from intragenic oligos (intra-exon probes) for each of the two fusion partners are indicative of the same fusion breakpoints as seen from the chimeric oligos. (D) The exact fusion breakpoint between *TCF3 *and *PBX1 *was confirmed by cDNA sequencing. (E) Chromosome banding and fluorescence *in situ *hybridization analyses of the same cell line demonstrated rearranged chromosomes from the translocation t(1;19)(q23;p13), which implicates the loci of *TCF3 *and *PBX1*.

**Figure 3:**
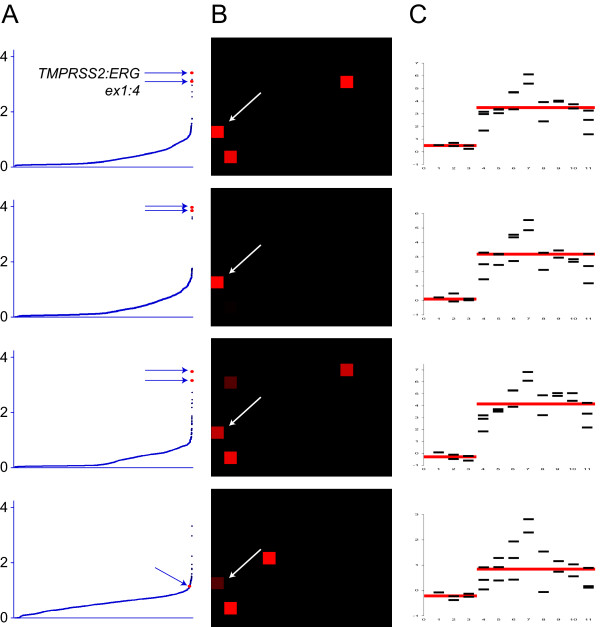
**Fusion gene plots for four individual prostate cancer samples with the same fusion event**. The four samples all had fusion transcripts with junctions between *TMPRSS2 *exon 1 and *ERG *exon 4. (A) Chimeric sequences plotted with increasing fusion scores. For the three first samples, the chimera of *TMPRSS2:ERG *exon1:4 had the highest ranking out of the 10,297 tested combinations. (B) Fusion map of each chimeric exon-exon junction of *TMPRSS2 *and *ERG*. Intensities of red indicate the relative values of the medians for the four replicate oligos for each chimeric exon-exon junction and the white arrows point to the correct fusion breakpoints. (C) Measurements from intragenic oligos (intra-exon probes) for *ERG *demonstrate a shift in intensities between exons three and four.

To combine the information from the chimeric junction measurements with that of the longitudinal intragenic profiles, a fusion score was calculated for all fusion transcripts and their respective breakpoints (details in Materials and Methods). This enabled an objective and automated evaluation of the presence of fusion genes, and the fusion score was calculated for 10,297 possible fusion events. The positive control fusion transcripts, with their correct breakpoint positions, was ranked as the number one hit in four out of the five samples run on the short-oligo microarray (Figures 2A and 3A), thus validating the concept. For prostate cancer sample P140, the expected *TMPRSS2 *exon 1:*ERG *exon 4 fusion gene was assigned a fusion score rank of 95 within the 10,297 fusion breakpoints (and number one within the 154 measured junctions of *TMPRSS2:ERG*). When dissecting the values behind the fusion score for this positive control, we see that the intensity of the chimeric oligo was particularly low. This is also in compliance with RT-PCR results from the prostate cancer samples, demonstrating that this sample had a low expression level of the fusion gene as compared to the other samples (data not shown).

To evaluate the top fusion score hits and positive control fusion genes further, we visualized them via two independent paths, using either the chimeric probe set (Figures 2B and 3B) or the longitudinal intragenic probe set (Figures 2C and 3C). The positive control fusion genes were clearly visualized for all six analyzed samples.

## Discussion

A novel microarray-based strategy is presented to screen for all known oncogenic fusion transcripts in a given sample, combining measurements of chimeric transcript junctions with exon-wise measurements of individual fusion partners. This provides a viable alternative to the existing cytogenetic and PCR-based methods for fusion gene detection, as it enables an objective and automated genome-wide analysis in which all known as well as predicted fusion genes are assessed without requiring any *a priori *knowledge as to the likelihood of the clinical or genetic diagnosis. Furthermore, the precise mapping information on the fusion breakpoint is given within every positive hit. Finally, the method is carried out in a single experiment and does not include prior sequence-specific amplification.

Because fusion breakpoints mainly map to intronic sequences, the resulting fusion transcripts will, after pre-mRNA processing, consist of whole exonic building blocks. In fact, more than 90% of the mapped fusion breakpoints are located in intronic sequences [20]. Thus, independently of the intra-intronic location of the breakpoints, a detection of all exon-exon junctions between two fusion gene partners would in principle provide specific detection of fusion transcripts.

To our knowledge, this is the first time chimeric oligos targeting fusion gene junctions have been used in combination with measurements of longitudinal profiles of the individual fusion partners. Furthermore, the earlier publications on fusion gene measurements by oligo microarrays have not attempted to be genome-wide, restricting their use to either a few pre-defined fusion junctions and fusion genes [10-13] or to the exclusive use of intragenic oligos [21]. Our pilot experiment alone included 68,861 oligos, and the current version of the NimbleGen microarray platform enables analysis of up to 2.1 million oligos on a single microarray slide. Thus, scaling up to include all known fusion genes, as well as sets of novel candidate fusion genes detected by high-throughput sequencing strategies, can easily be achieved with the same resolution level as the genes included in our pilot run.

Next-generation sequencing approaches are beginning to provide numerous new pairs of fusion genes in individual biological samples [6-9]. However, this methodology is not feasible for screening purposes on large clinical sample series. The current microarray-based approach is suitable for assessing whether members of this growing set of novel fusion transcripts (alongside with the already known fusion genes) are indeed pathogenetic players in the various subgroups of cancer.

The reported fusion gene detection platform can be used irrespective of the tumor type in question. Detection of certain fusion genes has direct diagnostic implications in many leukemias and sarcomas, whereas other fusion genes are more promiscuous and can be found in several different cancer types. An example of the latter is the karyotypically cryptic translocation t(12;15)(p13;q25), resulting in the *ETV6:NTRK3 *fusion gene, which occurs in histologically and developmentally completely disparate tumors such as kidney and breast tumors, infantile fibrosarcoma, and acute myeloid leukemia [22].

## Conclusion

We have developed a novel high throughput method for detection of fusion genes with potentially significant applications in cancer diagnostics. Also, for research applications, there is a clear potential for detection of putative fusion genes and discovery of already known fusion genes in new cancer types.

## List of abbreviations

FISH: fluorescence *in situ *hybridization; RT-PCR: reverse transcriptase polymerase chain reaction.

## Competing interests

A patent application has been filed for the fusion gene microarray methodology.

## Authors' contributions

RIS coordinated the project and drafted the manuscript. GOST designed and programmed algorithms for oligo design and data analysis. ME, GEL, FM, FRR, and NC performed laboratory analyses. MRT, SH, TR, and RAL supervised parts of the project, and contributed to design of the study and discussion of results. All authors contributed to the writing, and have read and approved the final manuscript.

## Supplementary Material

Additional File 1 **Supplementary table. Fusion genes included in the microarray design.** Click here for file

## References

[B1] Mitelman F, Johansson B, Mertens F (2004). Fusion genes and rearranged genes as a linear function of chromosome aberrations in cancer. Nat Genet.

[B2] Tomlins SA, Rhodes DR, Perner S, Dhanasekaran SM, Mehra R, Sun XW, Varambally S, Cao X, Tchinda J, Kuefer R, Lee C, Montie JE, Shah RB, Pienta KJ, Rubin MA, Chinnaiyan AM (2005). Recurrent fusion of *TMPRSS2 *and *ETS *transcription factor genes in prostate cancer. Science.

[B3] Helgeson BE, Tomlins SA, Shah N, Laxman B, Cao Q, Prensner JR, Cao X, Singla N, Montie JE, Varambally S, Mehra R, Chinnaiyan AM (2008). Characterization of TMPRSS2:ETV5 and SLC45A3:ETV5 gene fusions in prostate cancer. Cancer Res.

[B4] Teixeira MR (2006). Recurrent fusion oncogenes in carcinomas. Crit Rev Oncog.

[B5] Kumar-Sinha C, Tomlins SA, Chinnaiyan AM (2008). Recurrent gene fusions in prostate cancer. Nat Rev Cancer.

[B6] Ruan Y, Ooi HS, Choo SW, Chiu KP, Zhao XD, Srinivasan KG, Yao F, Choo CY, Liu J, Ariyaratne P, Bin WG, Kuznetsov VA, Shahab A, Sung WK, Bourque G, Palanisamy N, Wei CL (2007). Fusion transcripts and transcribed retrotransposed loci discovered through comprehensive transcriptome analysis using Paired-End diTags (PETs). Genome Res.

[B7] Chen W, Kalscheuer V, Tzschach A, Menzel C, Ullmann R, Schulz MH, Erdogan F, Li N, Kijas Z, Arkesteijn G, Pajares IL, Goetz-Sothmann M, Heinrich U, Rost I, Dufke A, Grasshoff U, Glaeser B, Vingron M, Ropers HH (2008). Mapping translocation breakpoints by next-generation sequencing. Genome Res.

[B8] Campbell PJ, Stephens PJ, Pleasance ED, O'meara S, Li H, Santarius T, Stebbings LA, Leroy C, Edkins S, Hardy C, Teague JW, Menzies A, Goodhead I, Turner DJ, Clee CM, Quail MA, Cox A, Brown C, Durbin R, Hurles ME, Edwards PA, Bignell GR, Stratton MR, Futreal PA (2008). Identification of somatically acquired rearrangements in cancer using genome-wide massively parallel paired-end sequencing. Nat Genet.

[B9] Maher CA, Kumar-Sinha C, Cao X, Kalyana-Sundaram S, Han B, Jing X, Sam L, Barrette T, Palanisamy N, Chinnaiyan AM (2009). Transcriptome sequencing to detect gene fusions in cancer. Nature.

[B10] Nasedkina T, Domer P, Zharinov V, Hoberg J, Lysov Y, Mirzabekov A (2002). Identification of chromosomal translocations in leukemias by hybridization with oligonucleotide microarrays. Haematologica.

[B11] Nasedkina TV, Zharinov VS, Isaeva EA, Mityaeva ON, Yurasov RN, Surzhikov SA, Turigin AY, Rubina AY, Karachunskii AI, Gartenhaus RB, Mirzabekov AD (2003). Clinical screening of gene rearrangements in childhood leukemia by using a multiplex polymerase chain reaction-microarray approach. Clin Cancer Res.

[B12] Shi RZ, Morrissey JM, Rowley JD (2003). Screening and quantification of multiple chromosome translocations in human leukemia. Clin Chem.

[B13] Lu Q, Nunez E, Lin C, Christensen K, Downs T, Carson DA, Wang-Rodriguez J, Liu YT (2008). A sensitive array-based assay for identifying multiple TMPRSS2:ERG fusion gene variants. Nucleic Acids Res.

[B14] Jack I, Seshadri R, Garson M, Michael P, Callen D, Zola H, Morley A (1986). RCH-ACV: a lymphoblastic leukemia cell line with chromosome translocation 1;19 and trisomy 8. Cancer Genet Cytogenet.

[B15] Rosenfeld C, Goutner A, Choquet C, Venuat AM, Kayibanda B, Pico JL, Greaves MF (1977). Phenotypic characterisation of a unique non-T, non-B acute lymphoblastic leukaemia cell line. Nature.

[B16] Matsuo Y, Drexler HG (1998). Establishment and characterization of human B cell precursor-leukemia cell lines. Leuk Res.

[B17] BioMart, Martview. Versions: Ensembl 48, release 43, Feb 2007 (Sanger), NCBI36.

[B18] Brazma A, Hingamp P, Quackenbush J, Sherlock G, Spellman P, Stoeckert C, Aach J, Ansorge W, Ball CA, Causton HC, Gaasterland T, Glenisson P, Holstege FC, Kim IF, Markowitz V, Matese JC, Parkinson H, Robinson A, Sarkans U, Schulze-Kremer S, Stewart J, Taylor R, Vilo J, Vingron M (2001). Minimum information about a microarray experiment (MIAME)-toward standards for microarray data. Nat Genet.

[B19] Cerveira N, Ribeiro FR, Peixoto A, Costa V, Henrique R, Jerónimo C, Teixeira MR (2006). *TMPRSS2-ERG *gene fusion causing *ERG *overexpression precedes chromosome copy number changes in prostate carcinomas and paired HGPIN lesions. Neoplasia.

[B20] Novo FJ, de Mendíbil IO, Vizmanos JL (2007). TICdb: a collection of mapped translocation breakpoints in cancer. BMC Genomics.

[B21] Jhavar S, Reid A, Clark J, Kote-Jarai Z, Christmas T, Thompson A, Woodhouse C, Ogden C, Fisher C, Corbishley C, De-Bono J, Eeles R, Brewer D, Cooper C (2008). Detection of TMPRSS2-ERG Translocations in Human Prostate Cancer by Expression Profiling Using GeneChip Human Exon 1.0 ST Arrays. J Mol Diagn.

[B22] Lannon CL, Sorensen PH (2005). ETV6-NTRK3: a chimeric protein tyrosine kinase with transformation activity in multiple cell lineages. Semin Cancer Biol.

