# Supplementary material for: A universal assay for detection of oncogenic fusion transcripts by oligo microarray analysis
Source: Mol Cancer. 2009 Jan 19;8:5. doi: 10.1186/1476-4598-8-5 (PMC2633275; doi:10.1186/1476-4598-8-5)
Supplement: Additional File 1 — Supplementary table. Fusion genes included in the microarray design. [file 1476-4598-8-5-S1.pdf]

Supplementary Table 1. Fusion genes included in the microarray design.

Upstream (5') fusion partner				Downstream (3') fusion partner			
Gene symbol	Ensembl ID	Cytoband	Bp from pter	Gene symbol	Ensembl ID	Cytoband	Bp from pter
AKAP9	ENSG00000127914	7q21.2	91 408 128	BRAF	ENSG00000157764	7q34	140 080 754
ARHGAP20	ENSG00000137727	11q23.2	109 952 976	BRWD3	ENSG00000165288	Xq13	79 813 009
ASPSCR1	ENSG00000169696	17q25.3	77 528 715	TFE3	ENSG00000068323	Xp11.23	48 771 186
ATIC	ENSG00000138363	2q35	215 885 036	ALK	ENSG00000171094	2p23.2	29 269 594
BCL11B	ENSG00000127152	14q32.2	98 705 377	TLX3	ENSG00000164438	5q35.1	170 668 893
BCL3	ENSG00000069399	19q13.31	49 943 820	MYC	ENSG00000136997	8q24.21	128 817 498
BCL7A	ENSG00000110987	12q24.31	120 944 244	MYC	ENSG00000136997	8q24.21	128 817 498
BCR	ENSG00000186716	22q11.23	21 852 552	ABL1	ENSG00000097007	9q34.12	132 579 089
BCR	ENSG00000186716	22q11.23	21 852 552	FGFR1	ENSG00000077782	8p12	38 389 406
BCR	ENSG00000186716	22q11.23	21 852 552	JAK2	ENSG00000096968	9p24.1	4 975 245
BCR	ENSG00000186716	22q11.23	21 852 552	PDGFRA	ENSG00000134853	4q12	54 790 204
BIRC3	ENSG00000023445	11q22.2	101 693 404	MALT1	ENSG00000172175	18q21.31	54 489 598
BRD4	ENSG00000141867	19p13.12	15 209 301	NUT	ENSG00000184507	15q14	32 425 358
BRWD3	ENSG00000165288	Xq13	79 813 009	ARHGAP20	ENSG00000137727	11q23.2	109 952 976
BTG1	ENSG00000133639	12q21.33	91 061 030	MYC	ENSG00000136997	8q24.21	128 817 498
CARS	ENSG00000110619	11p15.4	2 978 736	ALK	ENSG00000171094	2p23.2	29 269 594
CBFB	ENSG00000067955	16q22.1	65 620 551	MYH11	ENSG00000133392	16p13.11	15 704 495
CCDC6	ENSG00000108091	10q21.2	61 218 527	PDGFRB	ENSG00000113721	5q32	149 473 598
CCDC6	ENSG00000108091	10q21.2	61 218 527	RET	ENSG00000165731	10q11.21	42 892 528
CCND1	ENSG00000110092	11q13.3	69 165 054	FSTL3	ENSG00000070404	19p13.3	627 403
CDH11	ENSG00000140937	16q21	63 538 186	USP6	ENSG00000129204	17p13.2	4 972 411
CDK6	ENSG00000105810	7q21.2	92 072 175	EVI1	ENSG00000085276	3q26.2	170 283 999
CDK6	ENSG00000105810	7q21.2	92 072 175	MLL	ENSG00000118058	11q23.3	117 812 415
CDK6	ENSG00000105810	7q21.2	92 072 175	MLLT10	ENSG00000078403	10p12.31	21 863 100
CDK6	ENSG00000105810	7q21.2	92 072 175	TLX3	ENSG00000164438	5q35.1	170 668 893
CDKN2A	ENSG00000147889	9p21	21 957 751	CDKN2A	ENSG00000147889	9p21	21 957 751
CEP110	ENSG00000119397	9q33	122 876 962	FGFR1	ENSG00000077782	8p12	38 389 406
CHCHD7	ENSG00000170791	8q11.23	57 286 869	PLAG1	ENSG00000181690	8q12.1	57 236 037
CHIC2	ENSG00000109220	4q12	54 570 709	ETV6	ENSG00000139083	12p13.2	11 694 055

CIITA	ENSG00000179583	16p13	10 867 648	BCL6	ENSG00000113916	3q27.3	188 921 859
CLTC	ENSG00000141367	17q23.2	55 052 038	ALK	ENSG00000171094	2p23.2	29 269 594
CLTC	ENSG00000141367	17q23.2	55 052 038	TFE3	ENSG00000068323	Xp11.23	48 771 186
CNBP	ENSG00000169714	3p21.3	130 371 020	USP6	ENSG00000129204	17p13.2	4 972 411
COL1A1	ENSG00000108821	17q21.33	45 616 456	PDGFB	ENSG00000100311	22q13.1	37 949 310
COL1A1	ENSG00000108821	17q21.33	45 616 456	USP6	ENSG00000129204	17p13.2	4 972 411
COL1A2	ENSG00000164692	7q21.3	93 861 809	PLAG1	ENSG00000181690	8q12.1	57 236 037
CRTC1	ENSG00000105662	19p13	18 655 488	MAML2	ENSG00000184384	11q21	95 351 088
CTNNB1	ENSG00000168036	3p21	41 216 004	PLAG1	ENSG00000181690	8q12.1	57 236 037
EIF4A2	ENSG00000156976	3q27.3	187 984 060	BCL6	ENSG00000113916	3q27.3	188 921 859
EML1	ENSG00000066629	14q32	99 329 498	ABL1	ENSG00000097007	9q34.12	132 579 089
EPC1	ENSG00000120616	10p11	32 596 694	PHF1	ENSG00000112511	6p21.3	33 486 718
ERC1	ENSG00000082805	12p13.3	1 007 174	RET	ENSG00000165731	10q11.21	42 892 528
ETV6	ENSG00000139083	12p13.2	11 694 055	ABL1	ENSG00000097007	9q34.12	132 579 089
ETV6	ENSG00000139083	12p13.2	11 694 055	ABL2	ENSG00000143322	1q25.2	177 339 806
ETV6	ENSG00000139083	12p13.2	11 694 055	ACSL6	ENSG00000164398	5q31	131 170 735
ETV6	ENSG00000139083	12p13.2	11 694 055	ARNT	ENSG00000143437	1q21.2	149 048 810
ETV6	ENSG00000139083	12p13.2	11 694 055	CDX2	ENSG00000165556	13q12.2	27 434 273
ETV6	ENSG00000139083	12p13.2	11 694 055	EVI1	ENSG00000085276	3q26.2	170 283 999
ETV6	ENSG00000139083	12p13.2	11 694 055	FGFR3	ENSG00000068078	4p16.3	1 764 832
ETV6	ENSG00000139083	12p13.2	11 694 055	FLT3	ENSG00000122025	13q12	27 475 411
ETV6	ENSG00000139083	12p13.2	11 694 055	HLXB9	ENSG00000130675	7q36.3	156 490 326
ETV6	ENSG00000139083	12p13.2	11 694 055	JAK2	ENSG00000096968	9p24.1	4 975 245
ETV6	ENSG00000139083	12p13.2	11 694 055	MDS2	ENSG00000197880	1p36.12	23 826 411
ETV6	ENSG00000139083	12p13.2	11 694 055	MN1	ENSG00000169184	22q13	26 474 266
ETV6	ENSG00000139083	12p13.2	11 694 055	NTRK3	ENSG00000140538	15q25.3	86 221 026
ETV6	ENSG00000139083	12p13.2	11 694 055	PDGFRB	ENSG00000113721	5q32	149 473 598
ETV6	ENSG00000139083	12p13.2	11 694 055	PER1	ENSG00000179094	17p13.1	7 984 534
ETV6	ENSG00000139083	12p13.2	11 694 055	RUNX1	ENSG00000159216	21q22.12	35 081 975
ETV6	ENSG00000139083	12p13.2	11 694 055	SYK	ENSG00000165025	9q22.31	92 603 890
ETV6	ENSG00000139083	12p13.2	11 694 055	TCBA1	ENSG00000188580	6q22.33	124 166 985
ETV6	ENSG00000139083	12p13.2	11 694 055	TTL	ENSG00000114999	2q13	112 956 369
EWSR1	ENSG00000182944	22q12.2	27 994 283	ATF1	ENSG00000123268	12q13.12	49 444 128
EWSR1	ENSG00000182944	22q12.2	27 994 283	DDIT3	ENSG00000175197	12q13.3	56 196 640
EWSR1	ENSG00000182944	22q12.2	27 994 283	ERG	ENSG00000157554	21q22.2	38 675 671
EWSR1	ENSG00000182944	22q12.2	27 994 283	ETV1	ENSG00000006468	7p21.2	13 897 379

EWSR1	ENSG00000182944	22q12.2	27 994 283	ETV4	ENSG00000175832	17q21.31	38 960 738
EWSR1	ENSG00000182944	22q12.2	27 994 283	FEV	ENSG00000163497	2q35	219 554 053
EWSR1	ENSG00000182944	22q12.2	27 994 283	FLI1	ENSG00000151702	11q24.3	128 069 193
EWSR1	ENSG00000182944	22q12.2	27 994 283	NR4A3	ENSG00000119508	9q31.1	101 623 958
EWSR1	ENSG00000182944	22q12.2	27 994 283	POU5F1	ENSG00000204531	6p21.31	31 240 099
EWSR1	ENSG00000182944	22q12.2	27 994 283	TEC	ENSG00000135605	4p12	47 832 557
EWSR1	ENSG00000182944	22q12.2	27 994 283	WT1	ENSG00000184937	11p13	32 365 901
EWSR1	ENSG00000182944	22q12.2	27 994 283	ZNF278	ENSG00000100105	22q12.2	30 051 790
EWSR1	ENSG00000182944	22q12.2	27 994 283	ZNF384	ENSG00000126746	12p13.31	6 646 078
FGFR1OP	ENSG00000112486	6q27	167 332 660	FGFR1	ENSG00000077782	8p12	38 389 406
FGFR1OP2	ENSG00000111790	12p12.1	26 982 583	FGFR1	ENSG00000077782	8p12	38 389 406
FHIT	ENSG00000189283	3p14.2	59 712 992	HMGA2	ENSG00000149948	12q15	64 504 507
FIP1L1	ENSG00000145216	4q12	53 938 620	PDGFRA	ENSG00000134853	4q12	54 790 204
FLT3	ENSG00000122025	13q12	27 475 411	ETV6	ENSG00000139083	12p13.2	11 694 055
FUS	ENSG00000089280	16p11.2	31 098 954	ATF1	ENSG00000123268	12q13.12	49 444 128
FUS	ENSG00000089280	16p11.2	31 098 954	CREB3L1	ENSG00000157613	11q11	46 256 136
FUS	ENSG00000089280	16p11.2	31 098 954	CREB3L2	ENSG00000182158	7q34	137 210 267
FUS	ENSG00000089280	16p11.2	31 098 954	DDIT3	ENSG00000175197	12q13.3	56 196 640
FUS	ENSG00000089280	16p11.2	31 098 954	DDIT3	ENSG00000175197	12q13.3	56 196 640
FUS	ENSG00000089280	16p11.2	31 098 954	ERG	ENSG00000157554	21q22.2	38 675 671
GAPDH	ENSG00000111640	12p13	6 513 872	BCL6	ENSG00000113916	3q27.3	188 921 859
GOLGA5	ENSG00000066455	14q32.12	92 330 403	RET	ENSG00000165731	10q11.21	42 892 528
GOPC	ENSG00000047932	6q22.1	117 988 125	ROS1	ENSG00000047936	6q22.1	117 716 156
HAS2	ENSG00000170961	8q24.12	122 694 719	PLAG1	ENSG00000181690	8q12.1	57 236 037
HIP1	ENSG00000127946	7q11.23	75 001 345	PDGFRB	ENSG00000113721	5q32	149 473 598
HIST1H4I	ENSG00000198339	6p22.1	27 214 346	BCL6	ENSG00000113916	3q27.3	188 921 859
HMGA1	ENSG00000137309	6p21.31	34 312 628	LAMA4	ENSG00000112769	6q21	112 536 654
HMGA2	ENSG00000149948	12q15	64 504 507	CCNB1IP1	ENSG00000100814	14q11.2	19 849 370
HMGA2	ENSG00000149948	12q15	64 504 507	COX6C	ENSG00000164919	8q22.2	100 959 548
HMGA2	ENSG00000149948	12q15	64 504 507	CXCR7	ENSG00000144476	2q37.3	237 143 182
HMGA2	ENSG00000149948	12q15	64 504 507	FHIT	ENSG00000189283	3p14.2	59 712 992
HMGA2	ENSG00000149948	12q15	64 504 507	LHFP	ENSG00000183722	13q13.3	38 815 029
HMGA2	ENSG00000149948	12q15	64 504 507	LPP	ENSG00000145012	3q28	189 606 603
HMGA2	ENSG00000149948	12q15	64 504 507	RAD51L1	ENSG00000182185	14q24.1	67 360 014
HP	ENSG00000197711	16q22.1	70 646 009	MRPS10	ENSG00000048544	6	42 282 517
HSP90AA1	ENSG00000080824	14p12	101 617 139	BCL6	ENSG00000113916	3q27.3	188 921 859

HSP90AB1	ENSG00000096384	6p12	44 322 802	BCL6	ENSG00000113916	3q27.3	188 921 859
IKZF1	ENSG00000185811	7p12.2	50 314 924	BCL6	ENSG00000113916	3q27.3	188 921 859
IL2	ENSG00000109471	4q27	123 592 080	TNFRSF17	ENSG00000048462	16p13.13	11 966 465
IL21R	ENSG00000103522	16p12.1	27 321 224	BCL6	ENSG00000113916	3q27.3	188 921 859
ITK	ENSG00000113263	5q33.3	156 540 432	SYK	ENSG00000165025	9q22.31	92 603 890
JAZF1	ENSG00000153814	7p15	27 836 726	PHF1	ENSG00000112511	6p21.3	33 486 718
JAZF1	ENSG00000153814	7p15	27 836 726	SUZ12	ENSG00000178691	17q11.2	27 288 185
KIAA1509	ENSG00000015133	14q32.12	90 808 722	PDGFRA	ENSG00000134853	4q12	54 790 204
KIAA1618	ENSG00000180843	17q25.3	75 849 262	ALK	ENSG00000171094	2p23.2	29 269 594
KTN1	ENSG00000126777	14q22.3	55 116 777	RET	ENSG00000165731	10q11.21	42 892 528
LCP1	ENSG00000136167	13q14.13	45 598 059	BCL6	ENSG00000113916	3q27.3	188 921 859
LIFR	ENSG00000113594	5p13.1	38 510 823	PLAG1	ENSG00000181690	8q12.1	57 236 037
MALAT1	ENSG00000204691	11q13.1	65 023 092	TFEB	ENSG00000112561	6p21.1	41 759 694
MEF2D	ENSG00000116604	1q21	154 700 143	DAZAP1	ENSG00000071626	19p13.3	1 358 584
MLL	ENSG00000118058	11q23.3	117 812 415	ABI1	ENSG00000136754	10p11.2	27 075 528
MLL	ENSG00000118058	11q23.3	117 812 415	AFF1	ENSG00000172493	4q21	88 075 187
MLL	ENSG00000118058	11q23.3	117 812 415	AFF3	ENSG00000144218	2q11.2	99 530 150
MLL	ENSG00000118058	11q23.3	117 812 415	AFF4	ENSG00000072364	5q31	132 238 970
MLL	ENSG00000118058	11q23.3	117 812 415	ARHGAP26	ENSG00000145819	5q31	142 130 133
MLL	ENSG00000118058	11q23.3	117 812 415	ARHGEF12	ENSG00000196914	11q23.3	119 713 156
MLL	ENSG00000118058	11q23.3	117 812 415	CASC5	ENSG00000137812	15q14	38 673 755
MLL	ENSG00000118058	11q23.3	117 812 415	CBL	ENSG00000110395	11q23.3	118 582 200
MLL	ENSG00000118058	11q23.3	117 812 415	CELSR3	ENSG00000008300	3p21	48 637 835
MLL	ENSG00000118058	11q23.3	117 812 415	CLP1	ENSG00000172409	11q12	57 181 206
MLL	ENSG00000118058	11q23.3	117 812 415	CREBBP	ENSG00000005339	16p13.3	3 716 572
MLL	ENSG00000118058	11q23.3	117 812 415	CXXC6	ENSG00000138336	10q21	69 990 419
MLL	ENSG00000118058	11q23.3	117 812 415	DAB2IP	ENSG00000136848	9q33.1	123 368 983
MLL	ENSG00000118058	11q23.3	117 812 415	ELL	ENSG00000105656	19p13.11	18 414 475
MLL	ENSG00000118058	11q23.3	117 812 415	EP300	ENSG00000100393	22q13.2	39 817 736
MLL	ENSG00000118058	11q23.3	117 812 415	EPS15	ENSG00000085832	1p32.3	51 592 523
MLL	ENSG00000118058	11q23.3	117 812 415	FNBP1	ENSG00000187239	9q34.11	131 689 287
MLL	ENSG00000118058	11q23.3	117 812 415	FOXO3A	ENSG00000118689	6q21	108 987 731
MLL	ENSG00000118058	11q23.3	117 812 415	GAS7	ENSG00000007237	17p13.1	9 754 651
MLL	ENSG00000118058	11q23.3	117 812 415	GMPS	ENSG00000163655	3q25.31	157 071 019
MLL	ENSG00000118058	11q23.3	117 812 415	GPHN	ENSG00000171723	14q23.3	66 043 878
MLL	ENSG00000118058	11q23.3	117 812 415	LASP1	ENSG00000002834	17q12	34 279 894

MLL	ENSG00000118058	11q23.3	117 812 415	LPP	ENSG00000145012	3q28	189 606 603
MLL	ENSG00000118058	11q23.3	117 812 415	MAPRE1	ENSG00000101367	20q11.1	30 871 395
MLL	ENSG00000118058	11q23.3	117 812 415	MLL	ENSG00000118058	11q23.3	117 812 415
MLL	ENSG00000118058	11q23.3	117 812 415	MLLT1	ENSG00000130382	19p13.3	6 163 966
MLL	ENSG00000118058	11q23.3	117 812 415	MLLT10	ENSG00000078403	10p12.31	21 863 100
MLL	ENSG00000118058	11q23.3	117 812 415	MLLT11	ENSG00000143443	1q21	149 286 840
MLL	ENSG00000118058	11q23.3	117 812 415	MLLT3	ENSG00000171843	9p21.3	20 331 663
MLL	ENSG00000118058	11q23.3	117 812 415	MLLT4	ENSG00000130396	6q27	167 970 520
MLL	ENSG00000118058	11q23.3	117 812 415	MLLT6	ENSG00000108292	17q12	34 115 321
MLL	ENSG00000118058	11q23.3	117 812 415	MLLT7	ENSG00000184481	Xq13.1	70 232 772
MLL	ENSG00000118058	11q23.3	117 812 415	MYO1F	ENSG00000142347	19p13.3	8 491 689
MLL	ENSG00000118058	11q23.3	117 812 415	PICALM	ENSG00000073921	11q14.2	85 346 134
MLL	ENSG00000118058	11q23.3	117 812 415	RARA	ENSG00000131759	17q21.2	35 718 972
MLL	ENSG00000118058	11q23.3	117 812 415	RUNX1T1	ENSG00000079102	8q22	93 040 328
MLL	ENSG00000118058	11q23.3	117 812 415	SEPT11	ENSG00000138758	4q21	78 089 919
MLL	ENSG00000118058	11q23.3	117 812 415	SEPT2	ENSG00000168385	2q37	241 903 396
MLL	ENSG00000118058	11q23.3	117 812 415	SEPT5	ENSG00000184702	22q11.21	18 082 112
MLL	ENSG00000118058	11q23.3	117 812 415	SEPT6	ENSG00000125354	Xq24	118 633 715
MLL	ENSG00000118058	11q23.3	117 812 415	SEPT9	ENSG00000184640	17q25	72 909 732
MLL	ENSG00000118058	11q23.3	117 812 415	SH3GL1	ENSG00000141985	19p13.3	4 311 370
MLL	ENSG00000118058	11q23.3	117 812 415	SORBS2	ENSG00000154556	4q35.1	186 743 592
MLL	ENSG00000118058	11q23.3	117 812 415	ZFYVE19	ENSG00000166140	15q14	38 886 724
MSI2	ENSG00000153944	17q23.2	52 688 930	HOXA9	ENSG00000078399	7p15.2	27 168 583
MSN	ENSG00000147065	Xq12	64 804 236	ALK	ENSG00000171094	2p23.2	29 269 594
MYC	ENSG00000136997	8q24.21	128 817 498	BCL7A	ENSG00000110987	12q24.31	120 944 244
MYC	ENSG00000136997	8q24.21	128 817 498	BTG1	ENSG00000133639	12q21.33	91 061 030
MYH9	ENSG00000100345	22q12.3	35 007 273	ALK	ENSG00000171094	2p23.2	29 269 594
MYST3	ENSG00000083168	8p11	41 907 430	ASXL2	ENSG00000143970	2p24.1	25 814 065
MYST3	ENSG00000083168	8p11	41 907 430	CREBBP	ENSG00000005339	16p13.3	3 716 572
MYST3	ENSG00000083168	8p11	41 907 430	EP300	ENSG00000100393	22q13.2	39 817 736
MYST3	ENSG00000083168	8p11	41 907 430	NCOA2	ENSG00000140396	8q13.3	71 196 006
MYST4	ENSG00000156650	10q22.2	76 255 346	CREBBP	ENSG00000005339	16p13.3	3 716 572
NACA	ENSG00000196531	12q13.3	55 392 484	BCL6	ENSG00000113916	3q27.3	188 921 859
NCOA4	ENSG00000138293	10q11.23	51 235 233	RET	ENSG00000165731	10q11.21	42 892 528
NFKB2	ENSG00000077150	10q24.32	104 144 320	TBXAS1	ENSG00000059377	7q34	139 124 668
NIN	ENSG00000100503	14q22.1	50 256 232	PDGFRB	ENSG00000113721	5q32	149 473 598

NONO	ENSG00000147140	Xq13.1	70 420 158	TFE3	ENSG00000068323	Xp11.23	48 771 186
NPM1	ENSG00000181163	5q35.1	170 746 725	ALK	ENSG00000171094	2p23.2	29 269 594
NPM1	ENSG00000181163	5q35.1	170 746 725	MLF1	ENSG00000178053	3q25.32	159 771 677
NPM1	ENSG00000181163	5q35.1	170 746 725	RARA	ENSG00000131759	17q21.2	35 718 972
NUMA1	ENSG00000137497	11q13.4	71 391 559	RARA	ENSG00000131759	17q21.2	35 718 972
NUP214	ENSG00000126883	9q34.13	132 990 797	ABL1	ENSG00000097007	9q34.12	132 579 089
NUP214	ENSG00000126883	9q34.13	132 990 797	DEK	ENSG00000124795	6p22.3	18 332 392
NUP214	ENSG00000126883	9q34.13	132 990 797	SET	ENSG00000119335	9q34.11	130 485 844
NUP98	ENSG00000110713	11p15.4	3 652 817	ADD3	ENSG00000148700	10q25.2	111 755 552
NUP98	ENSG00000110713	11p15.4	3 652 817	CCDC28A	ENSG00000024862	6q23	139 136 339
NUP98	ENSG00000110713	11p15.4	3 652 817	DDX10	ENSG00000178105	11q22.3	108 041 014
NUP98	ENSG00000110713	11p15.4	3 652 817	HOXA11	ENSG00000005073	7p15.2	27 187 654
NUP98	ENSG00000110713	11p15.4	3 652 817	HOXA13	ENSG00000106031	7p15.2	27 203 024
NUP98	ENSG00000110713	11p15.4	3 652 817	HOXA9	ENSG00000078399	7p15.2	27 168 583
NUP98	ENSG00000110713	11p15.4	3 652 817	HOXC11	ENSG00000123388	12q13.13	52 653 196
NUP98	ENSG00000110713	11p15.4	3 652 817	HOXC13	ENSG00000123364	12q13.13	52 618 843
NUP98	ENSG00000110713	11p15.4	3 652 817	HOXD11	ENSG00000128713	2q31.1	176 680 330
NUP98	ENSG00000110713	11p15.4	3 652 817	HOXD13	ENSG00000128714	2q31.1	176 665 778
NUP98	ENSG00000110713	11p15.4	3 652 817	JARID1A	ENSG00000073614	12p11	263 249
NUP98	ENSG00000110713	11p15.4	3 652 817	NSD1	ENSG00000165671	5q35.3	176 494 711
NUP98	ENSG00000110713	11p15.4	3 652 817	PRRX1	ENSG00000116132	1q23	168 898 947
NUP98	ENSG00000110713	11p15.4	3 652 817	PRRX2	ENSG00000167157	9q34.1	131 467 741
NUP98	ENSG00000110713	11p15.4	3 652 817	PSIP1	ENSG00000164985	9p22.2	15 454 064
NUP98	ENSG00000110713	11p15.4	3 652 817	RAP1GDS1	ENSG00000138698	4q23	99 401 692
NUP98	ENSG00000110713	11p15.4	3 652 817	TOP1	ENSG00000198900	20q12	39 090 876
NUP98	ENSG00000110713	11p15.4	3 652 817	WHSC1L1	ENSG00000147548	8p12	38 251 717
NUT	ENSG00000184507	15q14	32 425 358	BRD4	ENSG00000141867	19p13.12	15 209 301
OMD	ENSG00000127083	9q22.31	94 216 348	USP6	ENSG00000129204	17p13.2	4 972 411
PAX3	ENSG00000135903	2q36.1	222 772 851	FOXO1	ENSG00000150907	13q14.11	40 027 801
PAX5	ENSG00000196092	9p13.2	36 823 272	ETV6	ENSG00000139083	12p13.2	11 694 055
PAX7	ENSG00000009709	1p36.13	18 830 087	FOXO1	ENSG00000150907	13q14.11	40 027 801
PAX8	ENSG00000125618	2q13	113 690 046	PPARG	ENSG00000132170	3p25.2	12 304 359
PCM1	ENSG00000078674	8p22	17 824 788	JAK2	ENSG00000096968	9p24.1	4 975 245
PCM1	ENSG00000078674	8p22	17 824 788	RET	ENSG00000165731	10q11.21	42 892 528
PDE4DIP	ENSG00000178104	1q21.1	143 562 784	PDGFRB	ENSG00000113721	5q32	149 473 598
PICALM	ENSG00000073921	11q14.2	85 346 134	MLLT10	ENSG00000078403	10p12.31	21 863 100

PIM1	ENSG00000137193	6p21.2	37 245 957	BCL6	ENSG00000113916	3q27.3	188 921 859
PML	ENSG00000140464	15q24.1	72 074 067	RARA	ENSG00000131759	17q21.2	35 718 972
POU2AF1	ENSG00000110777	11q23.1	110 728 210	BCL6	ENSG00000113916	3q27.3	188 921 859
PRCC	ENSG00000143294	1q23.1	155 003 898	TFE3	ENSG00000068323	Xp11.23	48 771 186
PRDM16	ENSG00000142611	1p36.32	2 975 621	EVI1	ENSG00000085276	3q26.2	170 283 999
PRKAR1A	ENSG00000108946	17q24.2	64 019 705	RET	ENSG00000165731	10q11.21	42 892 528
RABEP1	ENSG00000029725	17p13.2	5 126 506	PDGFRB	ENSG00000113721	5q32	149 473 598
RANBP2	ENSG00000153201	2q13	108 702 369	ALK	ENSG00000171094	2p23.2	29 269 594
RBM15	ENSG00000162775	1p13.3	110 682 651	MKL1	ENSG00000196588	22q13.1	39 136 252
RHOH	ENSG00000168421	4p13	39 874 965	BCL6	ENSG00000113916	3q27.3	188 921 859
RLF	ENSG00000117000	1p32	40 399 632	MYCL1	ENSG00000116990	1p34.3	40 133 685
RPN1	ENSG00000163902	3q21.3	129 821 503	EVI1	ENSG00000085276	3q26.2	170 283 999
RUNX1	ENSG00000159216	21q22.12	35 081 975	CBFA2T3	ENSG00000129993	16q24.3	87 468 768
RUNX1	ENSG00000159216	21q22.12	35 081 975	EVI1	ENSG00000085276	3q26.2	170 283 999
RUNX1	ENSG00000159216	21q22.12	35 081 975	MDS1	ENSG00000206115	3q26.2	170 581 663
RUNX1	ENSG00000159216	21q22.12	35 081 975	RPL22	ENSG00000116251	1p36.31	6 167 667
RUNX1	ENSG00000159216	21q22.12	35 081 975	RUNX1T1	ENSG00000079102	8q22	93 040 328
RUNX1	ENSG00000159216	21q22.12	35 081 975	SH3D19	ENSG00000109686	4q31.3	152 260 889
RUNX1	ENSG00000159216	21q22.12	35 081 975	USP42	ENSG00000106346	7p22.2	6 111 102
RUNX1	ENSG00000159216	21q22.12	35 081 975	YTHDF2	ENSG00000198492	1p35	28 935 858
RUNX1	ENSG00000159216	21q22.12	35 081 975	ZNF687	ENSG00000143373	1q21.2	149 520 655
SEC31A	ENSG00000138674	4q21.3	83 958 839	ALK	ENSG00000171094	2p23.2	29 269 594
SENP6	ENSG00000112701	6q13	76 367 945	TCBA1	ENSG00000188580	6q22.33	124 166 985
SFPQ	ENSG00000116560	1p34.3	35 414 571	TFE3	ENSG00000068323	Xp11.23	48 771 186
SFRS3	ENSG00000112081	6p21.31	36 670 123	BCL6	ENSG00000113916	3q27.3	188 921 859
SPECC1	ENSG00000128487	17p11.2	19 930 927	PDGFRB	ENSG00000113721	5q32	149 473 598
SS18	ENSG00000141380	18q11.2	21 850 217	SSX1	ENSG00000126752	Xp11.23	47 999 696
SS18	ENSG00000141380	18q11.2	21 850 217	SSX2	ENSG00000187754	Xp11.22	52 742 671
SS18	ENSG00000141380	18q11.2	21 850 217	SSX4	ENSG00000204645	Xp11.23	48 127 912
SS18L1	ENSG00000184402	20q13.33	60 152 217	SSX1	ENSG00000126752	Xp11.23	47 999 696
STAT5B	ENSG00000173757	17q11.2	37 604 722	RARA	ENSG00000131759	17q21.2	35 718 972
TAF15	ENSG00000172660	17q12	31 160 601	CHN1	ENSG00000128656	2q31.1	175 372 338
TAF15	ENSG00000172660	17q12	31 160 601	NR4A3	ENSG00000119508	9q31.1	101 623 958
TAF15	ENSG00000172660	17q12	31 160 601	TEC	ENSG00000135605	4p12	47 832 557
TAF15	ENSG00000172660	17q12	31 160 601	ZNF384	ENSG00000126746	12p13.31	6 646 078
TAL1	ENSG00000162367	1p33	47 454 550	STIL	ENSG00000123473	1p32	47 488 401

TCBA1	ENSG00000188580	6q22.33	124 166 985	ETV6	ENSG00000139083	12p13.2	11 694 055
TCEA1	ENSG00000187735	8q11.23	55 045 401	PLAG1	ENSG00000181690	8q12.1	57 236 037
TCF12	ENSG00000140262	15q21.3	54 998 125	NR4A3	ENSG00000119508	9q31.1	101 623 958
TCF12	ENSG00000140262	15q21.3	54 998 125	TEC	ENSG00000135605	4p12	47 832 557
TCF3	ENSG00000071564	19p13.3	1 561 964	HLF	ENSG00000108924	17q22	50 697 370
TCF3	ENSG00000071564	19p13.3	1 561 964	PBX1	ENSG00000185630	1q23.3	162 795 496
TCF3	ENSG00000071564	19p13.3	1 561 964	TFPT	ENSG00000105619	19q13	59 302 142
TFG	ENSG00000114354	3q12.2	101 910 850	ALK	ENSG00000171094	2p23.2	29 269 594
TFG	ENSG00000114354	3q12.2	101 910 850	NR4A3	ENSG00000119508	9q31.1	101 623 958
TFG	ENSG00000114354	3q12.2	101 910 850	NTRK1	ENSG00000198400	1q23.1	155 052 166
TFRC	ENSG00000072274	3q29	197 260 553	BCL6	ENSG00000113916	3q27.3	188 921 859
THRAP3	ENSG00000054118	1p34.3	36 462 604	USP6	ENSG00000129204	17p13.2	4 972 411
TIAF1	ENSG00000196535	17q11.2	24 424 663	FGFR1	ENSG00000077782	8p12	38 389 406
TMPRSS2	ENSG00000184012	21q22.3	41 758 351	ERG	ENSG00000157554	21q22.2	38 675 671
TMPRSS2	ENSG00000184012	21q22.3	41 758 351	ETV1	ENSG00000006468	7p21.2	13 897 379
TMPRSS2	ENSG00000184012	21q22.3	41 758 351	ETV4	ENSG00000175832	17q21.31	38 960 738
TP53BP1	ENSG00000067369	15q15	41 486 699	PDGFRB	ENSG00000113721	5q32	149 473 598
TPM3	ENSG00000143549	1q21.3	152 395 457	ALK	ENSG00000171094	2p23.2	29 269 594
TPM3	ENSG00000143549	1q21.3	152 395 457	NTRK1	ENSG00000198400	1q23.1	155 052 166
TPM3	ENSG00000143549	1q21.3	152 395 457	PDGFRB	ENSG00000113721	5q32	149 473 598
TPM4	ENSG00000167460	19p13.12	16 039 348	ALK	ENSG00000171094	2p23.2	29 269 594
TPR	ENSG00000047410	1q31.1	184 549 577	MET	ENSG00000105976	7q31	116 099 695
TPR	ENSG00000047410	1q31.1	184 549 577	NTRK1	ENSG00000198400	1q23.1	155 052 166
TRIM24	ENSG00000122779	7q32-34	137 795 619	FGFR1	ENSG00000077782	8p12	38 389 406
TRIM24	ENSG00000122779	7q32-34	137 795 619	RARA	ENSG00000131759	17q21.2	35 718 972
TRIM33	ENSG00000197323	1p13.2	114 736 922	RET	ENSG00000165731	10q11.21	42 892 528
TRIP11	ENSG00000100815	14q32.12	91 505 614	PDGFRB	ENSG00000113721	5q32	149 473 598
TTL	ENSG00000114999	2q13	112 956 369	ETV6	ENSG00000139083	12p13.2	11 694 055
ZBTB16	ENSG00000109906	11q23	113 435 525	RARA	ENSG00000131759	17q21.2	35 718 972
ZMYM2	ENSG00000121741	13q11	19 430 810	FGFR1	ENSG00000077782	8p12	38 389 406
